# Calcitonin-Secreting Neuroendocrine Tumor of the Lung: A Diagnostic Dilemma

**DOI:** 10.7759/cureus.83422

**Published:** 2025-05-03

**Authors:** Prashanth Kotla, Agnes I Udoh, Cecilia Clement, Jing He

**Affiliations:** 1 School of Medicine, University of Texas Medical Branch at Galveston, Galveston, USA; 2 Department of Pathology, University of Texas Medical Branch at Galveston, Galveston, USA

**Keywords:** atypical carcinoid, calcitonin, lung neuroendocrine tumor, medullary thyroid carcinoma, primary neuroendocrine tumor

## Abstract

Calcitonin is primarily secreted by parafollicular C cells of the thyroid, with significantly elevated levels serving as the primary serum marker for medullary thyroid carcinoma. However, ectopic calcitonin secretion, though rare, has been reported in neuroendocrine tumors arising in extra-thyroidal sites such as the lung, breast, prostate, liver, and thymus. This can pose diagnostic challenges due to the immunohistochemical similarity between ectopic calcitonin-secreting neuroendocrine tumors and medullary thyroid carcinoma, particularly in patients with coexisting thyroid lesions. Here, we present a rare case of a calcitonin-secreting neuroendocrine tumor of the lung, aiming to enhance diagnostic awareness and highlight the potential for elevated serum calcitonin due to ectopic secretion from an extrathyroidal source.

## Introduction

Calcitonin is a peptide primarily secreted by thyroid-residing parafollicular C cells, though C cells are also found in the lungs, thymus, small bowel, and parathyroid glands, where they may produce calcitonin [1,2]. Its physiological role includes inhibiting osteoclast activity and promoting renal excretion of phosphate and calcium [1]. Medullary thyroid carcinoma (MTC) arises from the uncontrolled proliferation of parafollicular C cells, leading to elevated serum calcitonin levels, which serve as a highly sensitive disease marker and prognostic indicator [3,4].

However, the specificity of calcitonin as an MTC marker is limited, as mild elevations can also occur in conditions such as pernicious anemia, kidney failure, sepsis, hyperparathyroidism-related hypercalcemia, and liver cirrhosis [1,3]. Additionally, calcitonin-producing neuroendocrine tumors (NETs) can arise in the lungs, thymus, bladder, adrenal glands, and ovaries [1,2], often resulting in significant calcitonin elevations similar to those seen in MTC [1]. Case reports have documented such tumors [2]. Examples include insulinomas, gastric carcinoids, pheochromocytomas, and small cell lung carcinomas, all of which have been associated with elevated serum calcitonin levels [5]. In pulmonary NETs, it is hypothesized that these malignancies may arise from neuroendocrine cells normally present in the lungs, which constitute around 1% of lung tissue and produce hormones such as calcitonin, chromogranin A, and serotonin [1]. However, the exact mechanisms underlying their malignant transformation remain unclear, and a definitive causal link has not been established [1]. Diagnosing these ectopic calcitonin-secreting NETs can be challenging due to their immunohistochemical resemblance to MTC [2]. In cases where a concurrent thyroid lesion is present, such tumors may be misdiagnosed as MTC, potentially resulting in unnecessary interventions like thyroidectomy [2].

Here, we report a case of a calcitonin-secreting NET of the lung. Awareness of extrathyroidal NETs as a source of elevated calcitonin is crucial to ensure accurate diagnosis. A comprehensive histological workup and imaging surveillance are essential for distinguishing these tumors from MTC and guiding appropriate treatment.

## Case presentation

A 60-year-old male with a medical history of type 2 diabetes, hypertension, hyperlipidemia, chronic kidney disease, coronary artery disease status post-implantable cardioverter defibrillator placement, two prior myocardial infarctions, congestive heart failure, and a significant smoking history presented with right-sided neck swelling and pain.

**Figure 1:**
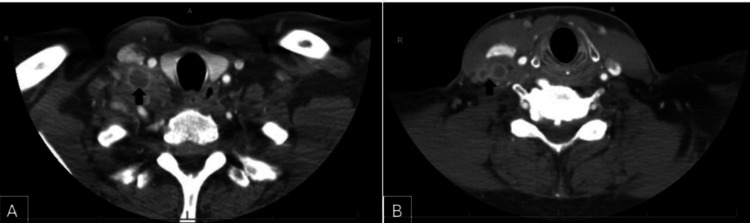
CT findings of the neck mass. CT images (A, B) show multiple necrotic lymph node conglomerates, indicated by black arrows.

A CT scan of the neck revealed multiple necrotic lymph nodes in the right supraclavicular and level III regions, with the largest conglomerate mass measuring 3.5 cm in the right supraclavicular area (Figures 1A, 1B).

**Figure 2:**
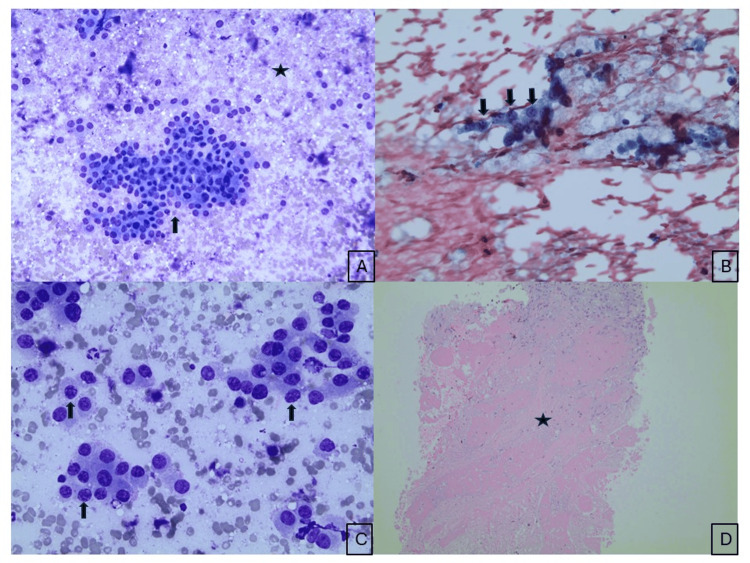
Cytological findings of the neck mass. Diff-Quik (A) and Papanicolaou-stained (B) cytologic smears showing high cellularity, consisting of loosely cohesive clusters and dispersed isolated oval to spindle-shaped cells with eccentric nuclei (black arrow). At higher magnification, the nuclei exhibited a finely granular “salt-and-pepper” chromatin pattern with small, indistinct nucleoli. The cells had a moderate amount of cytoplasm with indistinct cytoplasmic borders (black arrow) (C). Necrosis was observed in the cell block material (star) (D).

The patient underwent ultrasound-guided fine needle aspiration (FNA) of the neck mass. Cytologic evaluation of smears and the cell block demonstrated loosely clustered and singly dispersed neoplastic cells with round to elongated eccentrically placed nuclei, fine granular chromatin, inconspicuous nucleoli, and a moderate amount of cytoplasm. Mitotic activity and necrosis were also observed (Figures 2A-2D). 

A serum calcitonin test was performed, revealing an elevated level of 588 pg/mL (normal range: 0-7.5 pg/mL), raising suspicion for MTC. However, ultrasound of the thyroid showed a homogeneous thyroid with no distinct masses or lesions. Workup for potential RET mutation was negative.

**Figure 3:**
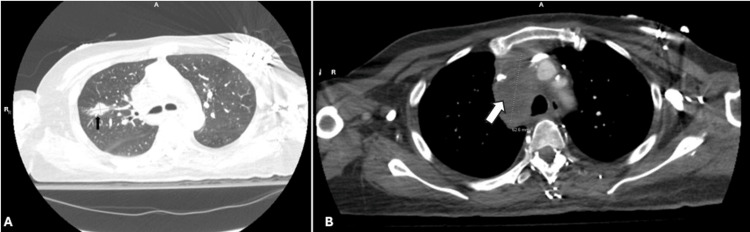
Chest CT findings. (A) An irregular nodule (black arrow) in the right upper lobe measuring 2.2 cm at its greatest dimension. (B) A hypodense mass (white arrow) in the superior mediastinum measuring approximately 6.3 cm at its greatest dimension.

Further imaging workup revealed a 2.2-cm spiculated mass in the right upper lobe of the lung (Figure 3), a conglomerate of necrotic right upper and lower paratracheal nodes measuring up to 6.3 cm, and an enlarged 17-mm subcarinal lymph node.

**Figure 4:**
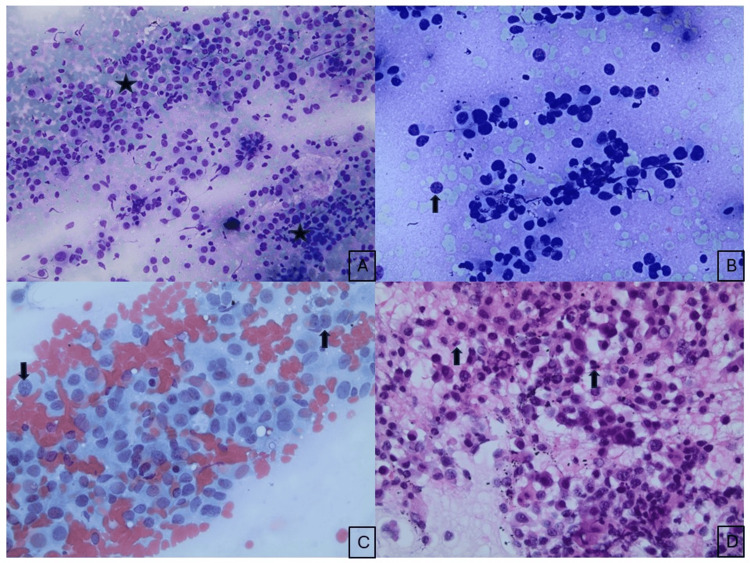
Cytological and histological findings of the EBUS-guided FNA and core biopsy of the lung mass. Diff-Quick (A, B) and Papanicolaou-stained (C) cytologic smears showing loosely cohesive clusters of neoplastic cells with round to oval nuclei, finely granular chromatin, small indistinct nucleoli, and moderate cytoplasm (black arrow and star), exhibiting similar cytomorphologic features to the neck mass. H&E staining of the cell block shows apoptotic bodies and mitotic activity (black arrow) (D). EBUS, endobronchial ultrasound; FNA, fine needle aspiration

While undergoing evaluation for multiple endocrine neoplasia type 2A with endocrinology, the need for a lung nodule biopsy was emphasized in the head and neck tumor board, given the absence of thyroid lesions or thyromegaly on ultrasound. Endobronchial ultrasound (EBUS)-guided FNA of the lung mass and the station 7 and 4R lymph nodes was performed, revealing tumor cells with similar morphological features. The cytological characteristics of the lung mass were consistent with those observed in the neck lymph node FNA (Figures 4A-4D).

**Figure 5:**
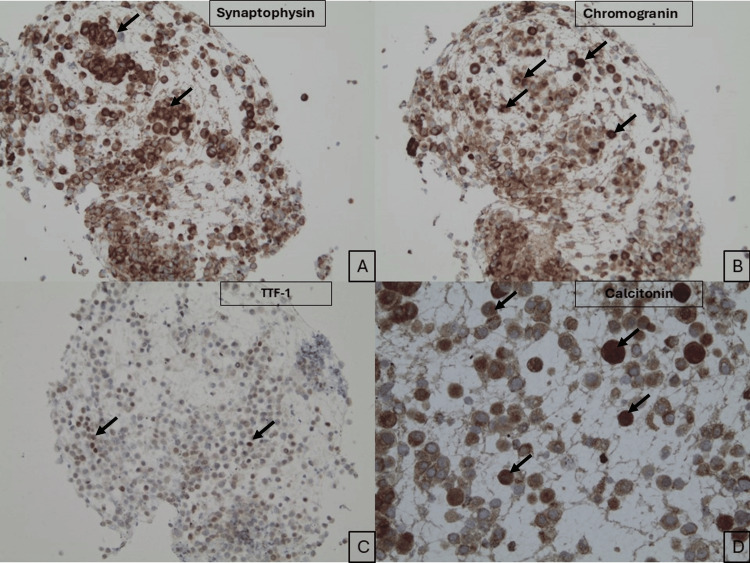
Immunostaining profile of the lung mass. (A) Synaptophysin immunostaining shows positive staining in the tumor cells (magnification: 200x). (B) Chromogranin immunostaining demonstrates strong positivity in the tumor cells (magnification: 200x). (C) Tumor cells exhibit nuclear expression of TTF-1 (magnification: 200x). (D) Calcitonin immunostaining shows strong positivity in the tumor cells (magnification: 200x) (black arrow).

Immunohistochemical profiling showed that tumor cells were positive for TTF-1, calcitonin, and neuroendocrine markers (chromogranin, synaptophysin, CD56) (Figures 5A-5D). Ki-67 immunostain showed a 60% tumor cell proliferation rate.

These results are most consistent with NET of the lung, favoring atypical carcinoid with nodal metastasis.

**Figure 6:**
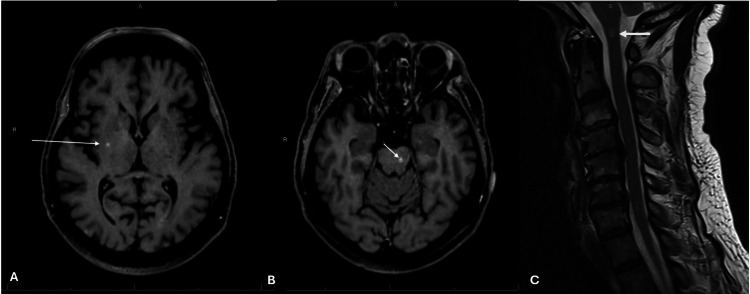
MRI findings of the brain and spine. (A) Brain MRI reveals scattered metastatic lesions (white arrow). (B) Post-chemotherapy MRI shows scattered brain metastases (white arrow). (C) Spinal MRI demonstrates stable metastatic lesions at the cervicomedullary junction (white arrow).

The clinical stage was determined to be stage 3B (cT1b, cN3, cM0). The patient was initially treated with vandetanib for two weeks, but treatment was discontinued due to a prolonged QTc interval observed on ECG. The patient received one dose of hormonal therapy with octreotide and later underwent four cycles of carboplatin and etoposide. During hospitalization for the first cycle of chemotherapy, an MRI of the head revealed findings concerning for multiple scattered metastatic lesions (Figure 6A). The patient was placed on dexamethasone, and a referral was made to radiation oncology. It was decided to monitor the lesions, as the MRI was performed shortly after the start of chemotherapy, and the patient was asymptomatic. A lesion near the spinal cord was also noted, and an MRI of the entire spine was planned for monitoring. As treatment progressed, the patient showed signs of response, maintaining the ability to perform activities of daily living. A follow-up brain MRI after completion of cycle 4 of carboplatin and etoposide showed multiple brain metastases (Figure 6B), indicating a mixed response to chemotherapy, along with some new lesions. Spinal MRI revealed stable metastatic lesions at the cervicomedullary junction (Figure 6C), and the decision was made to address this lesion after completing the current course of radiotherapy. The patient underwent a two-week course of whole brain radiotherapy.

At the follow-up with medical oncology, scanning was ordered for restaging. However, shortly after, the patient presented to the emergency department with altered mental status and generalized weakness. A subsequent neurological workup revealed a subacute left posterior cerebral artery stroke, likely secondary to a large left ventricular thrombus, as seen on CT of the head. The patient’s functional status progressively declined, and the family opted for hospice care, with palliative care being consulted. The patient was discharged to hospice and, unfortunately, passed away.

## Discussion

Differentiating between metastatic MTC and an extra-thyroidal NET with ectopic calcitonin secretion is critical, as it can significantly impact the treatment course. NETs of the lung are categorized into four groups: typical carcinoids, atypical carcinoids, large cell carcinomas, and small cell carcinomas [6]. Approximately 20% of primary lung lesions are attributed to NETs [7]. Among these, typical and atypical carcinoid tumors together account for roughly 2%, large cell NETs make up around 3%, and small cell NETs comprise the remaining 15% [7]. Immunohistochemical staining, combined with imaging and the overall clinical picture, plays a crucial role in diagnosis. The final pathology report for our patient diagnosed a calcitonin-secreting NET of the lung, with positive staining for chromogranin, synaptophysin, CD56, and TTF-1.

Chromogranin A is an acid-soluble protein stored in secretory granules found in various neuroendocrine cells, including those in the adrenal medulla and parathyroid chief cells, among other locations [8,9]. Synaptophysin is a membrane-associated synaptic vesicle protein that plays a role in modulating synaptic vesicle endocytosis [10]. Both markers are commonly used for identifying neuroendocrine neoplasms [10]. Positive staining is therefore expected in a range of NETs, including MTC and pulmonary carcinoids [10]. As anticipated, staining was positive in the lung biopsy sample, as noted above.

CD56, also known as neural cell adhesion molecule-1, is a membrane-bound glycoprotein that is prominently expressed in NETs, as well as in nerve cells and lymphocytes within tumor microenvironments [11]. CD56 is commonly used alongside chromogranin and synaptophysin to identify tumors with morphology indicative of neuroendocrine differentiation [12]. In a study by Kontogianni et al., which analyzed 20 small cell lung cancer samples characterized by extensive crush artifacts, all samples showed CD56 positivity in 75-100% of neoplastic cells [13].

TTF-1, or thyroid transcription factor-1, has been shown to be inconsistently expressed in lung carcinoid tumors [14]. In a study by Du et al., which analyzed 111 NETs (80 of pulmonary origin, 13 of thymic origin, 17 of pancreatic or GI origin, and 1 of ovarian origin), positive staining for TTF-1 was exclusively observed in NETs of pulmonary origin. TTF-1 expression was 100% specific, though not very sensitive, for identifying lung primary tumors in typical and atypical carcinoids, and possibly also in large cell neuroendocrine carcinomas [14]. Among the pulmonary NETs in this study, 14 were carcinoids (both typical and atypical), with 12 of the 14 that stained positive being located peripherally and exhibiting spindle cell morphology [14]. Our patient’s tumor, located in the right upper lobe, correlates with this peripheral localization, which aligns with the positive TTF-1 stain.

The cytological appearance of both the neck mass and lung mass specimens showed tumor cells arranged in loose clusters, with individual cells exhibiting a plasmacytoid appearance, eccentric nuclei, finely granular chromatin, inconspicuous nucleoli, and moderate cytoplasm. These cytological features are consistent with a neuroendocrine lesion. The positive staining for CEA and TTF-1, along with neuroendocrine markers such as synaptophysin, chromogranin, and CD56, raised the possibility of metastatic MTC. However, thyroid ultrasound did not reveal any identifiable thyroid lesions or thyromegaly. Given the positive calcitonin staining in the lung lesion, a diagnosis of calcitonin-secreting NET of the lung was made. This highlights the importance of a multidisciplinary approach that integrates immunohistochemistry, imaging, and clinical history to accurately identify extrathyroidal calcitonin-secreting NETs.

Additionally, the potential role of calcitonin expression in predicting the clinical course and prognosis of extra-thyroidal NETs warrants further investigation. In a study by Nozières et al., a retrospective analysis of 176 patients - primarily with pancreatic or bronchial NETs - examined serum calcitonin levels measured at least once over a 2.75-year period [15]. Elevated calcitonin levels (>100 ng/L) were significantly associated with tumors of pancreatic or bronchial origin (p < 0.0001) and with higher tumor grade (p = 0.006) [15]. Although high serum calcitonin was linked to poorer prognosis, this correlation did not remain statistically significant after multivariate analysis (p = 0.07) [15]. The authors suggest that the association between elevated calcitonin and poor prognosis may be confounded by its link to high-grade or poorly differentiated tumors, highlighting the need for further research into calcitonin’s prognostic value in neuroendocrine neoplasms [15].

Vahidi et al. report a case of a 69-year-old female in whom a left perihilar and lower lobe lung lesion was incidentally found on CT, performed due to left-sided abdominal pain [2]. A thyroid ultrasound revealed a left thyroid nodule measuring 0.9 cm. Subsequent EBUS FNA of the perihilar lesion revealed hypercellular cell clusters lacking cohesion, intermixed with spindle/oval-shaped cells with eccentric nuclei. Additional features included salt-and-pepper chromatin, inconspicuous small nuclei, and moderate cytoplasm. The tumor cells stained positive for chromogranin, synaptophysin, CD56, and calcitonin. The neuroendocrine histological findings raised suspicion for MTC, leading to a thyroid FNA, which identified a benign nodule. Serum calcitonin levels were within normal limits. A left lung wedge resection was performed, with morphological characteristics consistent with the initial EBUS FNA findings, leading to a diagnosis of calcitonin-secreting atypical carcinoid tumor. The authors emphasize the immunohistochemical similarities between MTC and extrathyroidal calcitonin-secreting NETs, noting that these tumors can arise in various organs, complicating accurate diagnosis. They also highlight that pulmonary neuroendocrine cells can release calcitonin in response to inflammatory or stress-induced states. The authors stress the importance of considering extrathyroidal NETs in the differential diagnosis when serum calcitonin levels are elevated in suspected MTC cases, as well as using thyroid imaging to distinguish between thyroid and extrathyroidal sources as the underlying etiology.

## Conclusions

While significantly elevated calcitonin levels are characteristic of MTC, particularly in the presence of a lesion with neuroendocrine morphology, it is essential to consider extrathyroidal calcitonin-secreting NETs. These tumors can arise in various extrathyroidal sites, and distinguishing them from MTC is crucial, as it can significantly impact the treatment approach. A comprehensive diagnostic workup, including thyroid imaging, immunohistochemical staining, and assessment of the overall clinical picture, is necessary to ensure an accurate diagnosis and appropriate management. The potential utility of elevated serum calcitonin in predicting the clinical course of calcitonin-secreting extra-thyroidal neoplasms warrants further investigation and may aid in optimizing treatment strategies for affected patients.

## References

[1] Llewellyn DC, Srirajaskanthan R, Vincent RP (2021). Calcitonin-secreting neuroendocrine neoplasms of the lung: a systematic review and narrative synthesis. Endocr Connect.

[2] Vahidi S, Stewart J, Amin K, Racila E, Li F (2018). Metastatic medullary thyroid carcinoma or calcitonin-secreting carcinoid tumor of lung? A diagnostic dilemma in a patient with lung mass and thyroid nodule. Diagn Cytopathol.

[3] Verbeek HH, de Groot JW, Sluiter WJ, Muller Kobold AC, van den Heuvel ER, Plukker JT, Links TP (2020). Calcitonin testing for detection of medullary thyroid cancer in people with thyroid nodules. Cochrane Database Syst Rev.

[4] Cai HJ, Wang H, Cao N (2020). Calcitonin-negative neuroendocrine tumor of the thyroid with metastasis to liver-rare presentation of an unusual tumor: a case report and review of literature. World J Clin Cases.

[5] Toledo SP, Lourenço DM Jr, Santos MA, Tavares MR, Toledo RA, Correia-Deur JE (2009). Hypercalcitoninemia is not pathognomonic of medullary thyroid carcinoma. Clinics (Sao Paulo).

[6] Hendifar AE, Marchevsky AM, Tuli R (2017). Neuroendocrine tumors of the lung: current challenges and advances in the diagnosis and management of well-differentiated disease. J Thorac Oncol.

[7] Rekhtman N (2022). Lung neuroendocrine neoplasms: recent progress and persistent challenges. Mod Pathol.

[8] D'amico MA, Ghinassi B, Izzicupo P, Manzoli L, Di Baldassarre A (2014). Biological function and clinical relevance of chromogranin A and derived peptides. Endocr Connect.

[9] Tomita T (2021). Significance of chromogranin A and synaptophysin in medullary thyroid carcinoma. Bosn J Basic Med Sci.

[10] Uhlig R, Dum D, Gorbokon N (2022). Synaptophysin and chromogranin A expression analysis in human tumors. Mol Cell Endocrinol.

[11] Jian Y, Zhang L, Gong L (2023). CD56 polysialylation promotes the tumorigenesis and progression via the Hedgehog and Wnt/β-catenin signaling pathways in clear cell renal cell carcinoma. Cancer Cell Int.

[12] Travis WD (2010). Advances in neuroendocrine lung tumors. Ann Oncol.

[13] Kontogianni K, Nicholson AG, Butcher D, Sheppard MN (2005). CD56: a useful tool for the diagnosis of small cell lung carcinomas on biopsies with extensive crush artefact. J Clin Pathol.

[14] Du EZ, Goldstraw P, Zacharias J (2004). TTF-1 expression is specific for lung primary in typical and atypical carcinoids: TTF-1-positive carcinoids are predominantly in peripheral location. Hum Pathol.

[15] Nozières C, Chardon L, Goichot B (2016). Neuroendocrine tumors producing calcitonin: characteristics, prognosis and potential interest of calcitonin monitoring during follow-up. Eur J Endocrinol.

